# Retraction Note: HDAC1-Mediated MicroRNA-124-5p Regulates NPY to Affect Learning and Memory Abilities in Rats with Depression

**DOI:** 10.1186/s11671-023-03827-3

**Published:** 2023-03-23

**Authors:** Chunling Tang, Jian Hu

**Affiliations:** Department of Psychiatry, The First Affiliated Hospital of Harbin Medical University, 23 Youzheng Street, Nangang District, Harbin, 150001 Heilongjiang China

**Retraction note: Nanoscale Res Lett (2021) 16:28** https://doi.org/10.1186/s11671-021-03477-3

The Editors in Chief have retracted this article. After publication, concerns were raised about the appearance of Western blots and the data in Table 1 that indicates that miR-124-3p may have been the target of the assays instead of miR-124-5p. The authors were unable to provide uncropped gels and blots and stated that some blots have been modified for "aesthetics " reasons. The Editors, therefore, have lost confidence in the integrity of the article's findings. Jian Hu agrees with this retraction. Chunling Tang did not respond to correspondence from the Editor about this retraction.

